# Recombinant Probiotic Expressing *Listeria* Adhesion Protein Attenuates *Listeria monocytogenes* Virulence *In Vitro*

**DOI:** 10.1371/journal.pone.0029277

**Published:** 2012-01-03

**Authors:** Ok Kyung Koo, Mary Anne Roshni Amalaradjou, Arun K. Bhunia

**Affiliations:** 1 Molecular Food Microbiology Laboratory, Department of Food Science, Purdue University, West Lafayette, Indiana, United States of America; 2 Department of Comparative Pathobiology, Purdue University, West Lafayette, Indiana, United States of America; Instituto Butantan, Brazil

## Abstract

**Background:**

*Listeria monocytogenes*, an intracellular foodborne pathogen, infects immunocompromised hosts. The primary route of transmission is through contaminated food. In the gastrointestinal tract, it traverses the epithelial barrier through intracellular or paracellular routes. Strategies to prevent *L. monocytogenes* entry can potentially minimize infection in high-risk populations. *Listeria* adhesion protein (LAP) aids *L. monocytogenes* in crossing epithelial barriers via the paracellular route. The use of recombinant probiotic bacteria expressing LAP would aid targeted clearance of *Listeria* from the gut and protect high-risk populations from infection.

**Methodology/Principal Findings:**

The objective was to investigate the ability of probiotic bacteria or LAP-expressing recombinant probiotic *Lactobacillus paracasei* (Lbp^LAP^) to prevent *L. monocytogenes* adhesion, invasion, and transwell-based transepithelial translocation in a Caco-2 cell culture model. Several wild type probiotic bacteria showed strong adhesion to Caco-2 cells but none effectively prevented *L. monocytogenes* infection. Pre-exposure to Lbp^LAP^ for 1, 4, 15, or 24 h significantly (*P*<0.05) reduced adhesion, invasion, and transepithelial translocation of *L. monocytogenes* in Caco-2 cells, whereas pre-exposure to parental *Lb. paracasei* had no significant effect. Similarly, Lbp^LAP^ pre-exposure reduced *L. monocytogenes* translocation by as much as 46% after 24 h. Lbp^LAP^ also prevented *L. monocytogenes*-mediated cell damage and compromise of tight junction integrity. Furthermore, Lbp^LAP^ cells reduced *L. monocytogenes*-mediated cell cytotoxicity by 99.8% after 1 h and 79% after 24 h.

**Conclusions/Significance:**

Wild type probiotic bacteria were unable to prevent *L. monocytogenes* infection *in vitro*. In contrast, Lbp^LAP^ blocked adhesion, invasion, and translocation of *L. monocytogenes* by interacting with host cell receptor Hsp60, thereby protecting cells from infection. These data show promise for the use of recombinant probiotics in preventing *L. monocytogenes* infection in high-risk populations.

## Introduction

*Listeria monocytogenes* causes a severe systemic infection (listeriosis) and poses a significant health risk to pregnant women, newborns, the elderly, and other immunocompromised individuals [1]. Annually, about 2,500 Americans contract invasive listeriosis with a mortality rate of 20–30% [2]. Traditional vaccination is not economical for the treatment and control of listeriosis owing to the small number of cases. Given increasing concerns about antibiotic resistance, the emergence of “superbugs” [3]–[5], and the lack of targeted treatments, recombinant lactobacilli expressing the genes required for pathogen adhesion and colonization, such as *Listeria* adhesion protein (LAP), might selectively prevent infection because adhesion and colonization are primary and crucial steps in pathogenesis [6]. A need exists for novel and effective strategies to prevent listeriosis in susceptible populations, but it requires a better understanding of the intestinal phase of *L. monocytogenes* infection.

Listeriosis is predominantly contracted through contaminated food, although neonatal listeriosis is acquired from the mother. During the gastrointestinal phase of infection, intestinal epithelial cells and M (microfold) cells are the primary sites of interaction. Adhesion, invasion, and translocation across the intestinal epithelial barrier are a prerequisite for pathogenesis [6]–[8]. Therefore, devising strategies to block the initial site of pathogen interaction is an effective and logical approach to protecting hosts against enteric infections [9], [10].

LAP is an alcohol acetaldehyde dehydrogenase (*lmo*1634), a housekeeping enzyme with a molecular mass of about 104 kDa [11]. It interacts strongly with host cells of intestinal origin [12], [13] and binds to host cell receptor Hsp60 [14]. More specifically, the N2 domain (Gly_224_ – Gly_411_) in the N-terminus of LAP interacts with Hsp60 [15]. Surface expression and secretion of LAP depend on SecA2, an auxiliary secretion system present in gram-positive bacteria [11], [16]. Our previous studies have demonstrated that LAP expression is enhanced in oxygen- and nutrient-limited conditions and at elevated temperatures (37–42°C) [16]–[18]. In the intestine, *L. monocytogenes* crosses the epithelial barrier by invading epithelial cells through the intracellular route using Internalin (InlA or InlB) proteins [19], [20]. Our recent data have, for the first time, shown that *Listeria* can also cross the epithelial barrier via the paracellular route [21]. Interaction of LAP with Hsp60 compromises the tight junction barrier, allowing increased paracellular translocation of *L. monocytogenes*. Furthermore, *L. monocytogenes* translocation occurs independently of InlA: a Δ*inl*A mutant strain translocated efficiently through the epithelial barrier [21].

Probiotic bacteria have long been used to promote human health [22]. These bacteria colonize and proliferate in the intestine, producing metabolites and macromolecules with beneficial effects including health maintenance and prevention or alleviation of enteric infection, allergic diseases, and chronic inflammatory diseases such as inflammatory bowel disease, Crohn's disease, and ulcerative colitis [3], [4], [23]–[25]. The use of probiotics to prevent and treat infections is gaining attention as a substitute for antibiotic or anti-inflammatory drugs because antibiotic resistance [26] and the emergence of “superbugs” threaten public health [3]–[5]. One of the most critical functions of probiotics is infection prevention, likely mediated by increased defensin production, induction of anti-inflammatory responses, suppression of pro-inflammatory cytokines (i.e., tumor necrosis factor α, interleukin [IL]-8, IL-6), increased production of short-chain fatty acids (butyrate) during fermentation, and improved epithelial tight junction barrier function [27]–[31]. Probiotic bacteria attach to intestinal cells via electrostatic or hydrophobic interactions, steric forces, lipoteichoic acids, or specific surface proteins [24], [32] and prevent pathogen binding through a mechanism referred to as steric hindrance [33], [34]. Probiotic bacterial cells [35], cell wall components such as S-layer proteins [27], and secretory compounds are also known to prevent enteric pathogen colonization [36] and neutralize toxins [37]. Although many enteric diseases have been controlled by probiotics, the approach has had limited success or been ineffective with *L. monocytogenes* [3], [33]. Furthermore, the normal anti-pathogen adhesive activity of probiotics is often unpredictable and unsatisfactory and may be unsuitable for inhibiting the attachment of specific pathogens to a host.

Genetic modification of probiotic bacteria to respond to target pathogens and toxins or to deliver biologics such as anti-inflammatory cytokines has become highly attractive [38]–[41]. Probiotic bacteria are generally regarded as safe and widely used in commercial probiotic products. Genetic modification allows for targeted pathogen elimination. For example, probiotic bacteria have been engineered to prevent *E. coli* heat-labile toxin [42] and cholera toxin binding to host receptors [43], *Helicobacter pylori* infection by expressing urease B [44], and HIV infection by expressing HIV-specific CD4 receptors [45] and to control *Salmonella enterica* infection by expressing flagellar antigen [46].

The objective of this study was to develop a recombinant probiotic strain expressing LAP to exclude adhesion, transepithelial translocation, and cell cytotoxicity of *L. monocytogenes* competitively in a cell culture model. We expected that a genetically engineered probiotic would exert its antimicrobial effect against the target pathogen directly through the expression of a foreign gene and indirectly through beneficial properties inherent in probiotics.

## Results

### Lactobacilli Showed Highest Attachment to Caco-2 Cells

**Figure 1:**
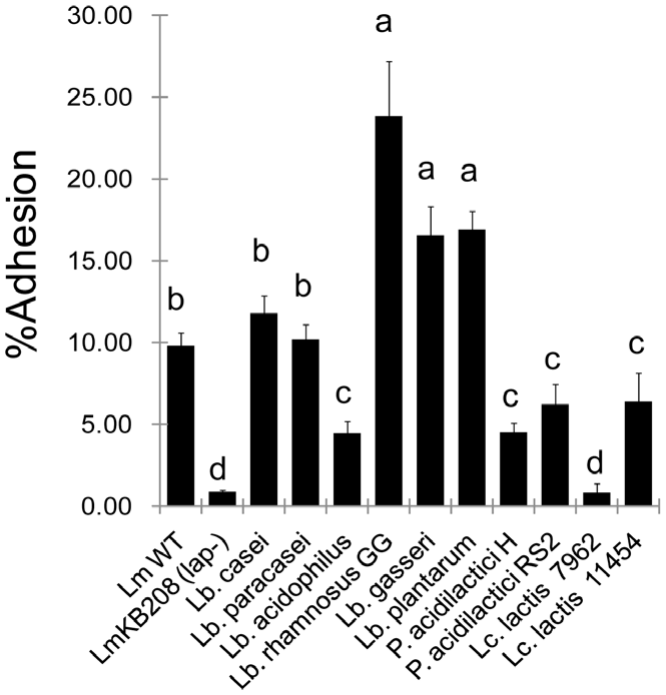
Adhesion profile of lactic acid bacteria to Caco-2 cells. Bacteria were added to Caco-2 cells at a ratio of 10:1. Percent adhesion was calculated relative to the inoculums that were added to the Caco-2 cells for the adhesion assay. The data are average (SD) of two independent experiments performed in triplicate. Bars marked with letters (a, b, c, d) are significantly different at *P*<0.05.

The ability to adhere to or colonize epithelial cells is an essential and prerequisite trait for probiotic bacteria [47], [48]. To select the most suitable candidate for genetic modification, we screened the attachment profiles of several lactic acid bacteria (LAB), including some well-characterized probiotics (*Lactobacillus* spp.) and bacteriocin-producing strains (*Pediococcus* and *Lactococcus*) to Caco-2 cells (Table 1; Fig 1). As reference controls, adhesion of *L. monocytogenes* F4244 (wild type [WT]) and the *lap*-deficient isogenic strain KB208 were also analyzed, showing about 9.78% and 0.84% adhesion, respectively. Attachment of LAB to Caco-2 cells varied from 0.78% to 23.8% with *Lactobacillus rhamnosus* showing the highest (23.8%) adhesion, followed by *Lb. plantarum* (16.9%), *Lb. gasseri* (16.6%), *Lb. casei* (11.8%), and *Lb. paracasei* (10.2%). *Lb. acidophilus*, *Pediococcus*, and *Lactococcus* attached to Caco-2 cells in significantly lower numbers than those of *L. monocytogenes* (*P*<0.0001). From this study, a representative strain of highly adherent *Lb. rhamnosus*, moderately high *Lb. paracasei*, and low-adherent *Lb. acidophilus* were chosen for subsequent experiments.

**Table 1:** Bacterial strains and plasmids.

Bacteria/plasmids	Strains	Description	Source
*Listeria monocytogenes*	F4244	Wild type, serotype 4b	Our collection
*L. monocytogenes*	KB208	F4244, LAP deficient strain (Em^R^ 5 µg/mL)	Our laboratory
*L. innocua*	F4248	Wild type	Our collection
*Lactobacillus acidophilus*	NRRL B1910	Wild type	Our collection
*Lb. casei*	KCTC 3109	Wild type	KCTC
*Lb. gasseri*	ATCC19992	Wild type	ATCC
*Lb. paracasei*	DUP13076	Wild type	Lactrys Biopharmaceuticals BV (Netherlands)
*Lb. paracasei*	LAP^+^ (AKB901)	*Lb. paracasei* expressing LAP of *L. monocytogenes* (Em^R^ 2 µg/ml)	This study
*Lb. paracasei* *Lb. plantarum*	LAP^-^NCDO	*Lb. paracasei* carrying control plasmid with no insert (Em^R^ 2 µg/ml)Wild type	This studyNCDO
*Lb. rhamnosus* GG	ATCC53103	Wild type	ATCC
*Pediococcus acidilactici*	H	Pediocin AcH-producing strain, Wild type	B. Ray (University of Wyoming)
*Ped. acidilactici*	RS2	Pediocin RS2-producing strain; Wild type	Our collection
*Lactococcus lactis*	ATCC 7962	Wild type	ATCC
*Lac. lactis*	ATCC 11454	Nisin-producing strain; Wild type	ATCC
Plasmids			
pGEM-T easy		Cloning vector (Am^R^ 50 µg/mL)	Promega
pGEM-LAPLm		pGEM-Teasy carrying *lap* of *L. monocytogenes*	This study
pLP401T		Expression vector for *Lactobacillus*, (Am^R^ 50 µg/mL and Em^R^ 2 µg/mL)	[49]
pLP401-LAP		pLP401 carrying *lap* of *L. monocytogenes*	This study

KCTC, Korean Type Culture Collection; ATCC, American Type Culture Collection; NCDO, National Collection of Dairy Organisms.

### Wild Type Lactobacilli Did Not Reduce *L. monocytogenes* Infection in Caco-2 Cells

We used three experimental approaches to examine whether the selected lactobacilli would reduce *L*. *monocytogenes* adhesion to Caco-2 cells, [10]: competitive exclusion, inhibition of adhesion, and displacement (Fig S1). Surprisingly, none of the lactobacilli reduced the adhesion of *L. monocytogenes* at significant levels regardless of method used (Fig S1), despite their uniform attachment to Caco-2 cells throughout the study. Five additional LAB strains also did not displace attached *L. monocytogenes* from Caco-2 cells (Fig S2a). We examined whether increased concentrations of lactobacilli could reduce *L*. *monocytogenes* adhesion. Lactobacilli added in 100-fold greater numbers also failed to displace attached *L. monocytogenes* (Fig S2b). These data clearly indicated that lactobacilli and other tested LAB strains were unable to reduce or prevent *L. monocytogenes* adhesion or colonization on epithelial cell surfaces, even in higher numbers.

### LAP of *L. monocytogenes* Cloning and Expression in *Lb. paracasei*

**Figure 2:**
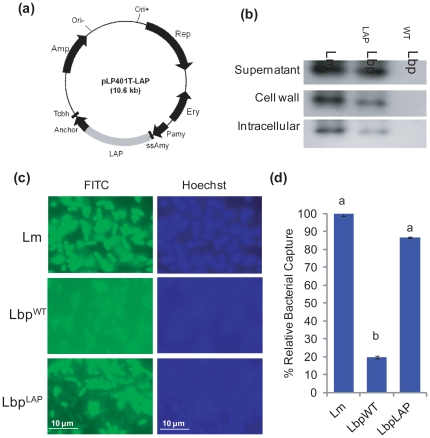
*Listeria* adhesion protein (LAP) expression analysis in recombinant *Lactobacillus paracasei* (Lbp^LAP^). (a) Plasmid map (10.6 kb) of LAP expression vector pLP401T (9.8 kb)-LAP (2.6 kb) [50]. **Ery**, erythromycin resistance gene; **Amp**, ampicillin resistance gene; Ori+ = origin of replication of *E. coli*, Ori- =  origin of replication of *Lactobacillus*; **LAP**, *Listeria* adhesion protein; **Pamy**, α-amylase promoter gene; **ssAmy**, secretion signal (36 aa) and the N-terminus (26 aa) of α-amylase gene; **Anchor**, anchor peptide (117 aa) gene of *Lb. casei*; **Tcbh**, transcription terminator of the cbh (conjugated bile acid hydrolase) gene; **Rep**, *repA* gene. (b) Western blot showing LAP expression in the supernatant, cell wall, and intracellular fractions of *Listeria monocytogenes* (Lm) and Lbp^LAP^ but absent in wild type *Lb. paracasei* (Lbp^WT^). Molecular weight of LAP from Lm and the recombinant Lbp^LAP^ was similar. (c) Immunofluorescence staining of bacteria (magnification 1000×) with anti-LAP MAb-H7 and fluorescein isothiocyanate-conjugated second antibody (left panel) and Hoechst dye (blue; right panel). Lbp^LAP^ and Lm (control) cells indicated the presence of surface-expressed LAP (green) that was absent in Lbp^WT^. (d) Binding (capture) of recombinant *Lb. paracasei* cells to paramagnetic beads coated with Hsp60 relative to *L. monocytogenes* (considered 100%). The data are average (SD) of two independent experiments performed in duplicate. Letters (a, b) indicate significant difference at *P*<0.05.

Because none of the WT LAB showed any discernable inhibition of *L. monocytogenes*, we sought to determine whether LAP expression in probiotic bacteria would reduce *L. monocytogenes* infection in the competitive exclusion experiments. We first cloned the *lap* gene in a *Lactobacillus* expression vector, pLP401-T [49], [50] (Fig 2a), and transformed it into *Lb. paracasei* (Table 1), which had an intermediate level of attachment to Caco-2 cells (see Fig 1; Note: the pLP401-T vector was originally designed for heterologous gene expression in *Lb. paracasei* and *Lb. casei*, hence we used *Lb. paracasei* to express *L. monocytogenes* LAP). Protein expression in recombinant *Lb. paracasei* (Lbp^LAP^) cell fractions was analyzed with Western blot. Data indicated that LAP was present in the supernatant (SN), cell wall (CW), and intracellular fractions (Fig 2b). Aminopeptidase C (PepC) assay confirmed that the SN and CW fraction had no apparent contamination from intracellular proteins (data not shown). Furthermore, anti-LAP MAb EM-H7 showed no reaction with protein bands from *Lb. paracasei* WT (Lbp^WT^) (see Fig 2b). These data indicated that LAP is surface associated in Lbp^LAP^ cells and would be available for interaction with mammalian cells. Additionally, immunofluorescence staining using anti-LAP MAb confirmed the surface localization (Fig 2c). LAP interacts with mammalian protein receptor Hsp60 [14]. To verify whether surface-expressed LAP from Lbp^LAP^ would interact with Hsp60, purified mammalian Hsp60 protein was immobilized on paramagnetic beads [51], and the capture rate of Lbp^LAP^ cells was determined relative to *L. monocytogenes* capture. If the bead-based capture efficiency of *L*. *monocytogenes* WT was considered 100%, the percent relative capture for Lbp^LAP^ cells was 86.5%, which was 4.4-fold higher than that of Lbp^WT^ (19.6%; Fig 2d). In a separate experiment, we also showed that pretreatment of Caco-2 cells with anti-Hsp60 monoclonal antibody (1 µg/ml) [14], [21] affected Lbp^LAP^ binding and subsequently *L. monocytogenes* adhesion (Fig S4). Collectively, these data confirmed that LAP of *L. monocytogenes* was successfully expressed in *Lb. paracasei* and surface-associated LAP efficiently interacted with Hsp60.

### Lbp^LAP^ Adherence and Translocation through Caco-2 Cell Monolayers

**Figure 3:**
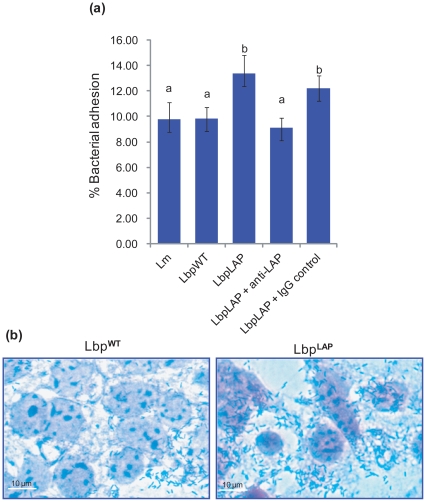
Adhesion characteristics of recombinant *Lactobacillus paracasei* (Lbp^LAP^) to Caco-2 cells. (a) Adhesion of Lbp^LAP^ was compared with wild type *Lb. paracasei* (Lbp^WT^) and *L. monocytogenes* (Lm). Bacterial cells were incubated with anti-LAP MAb-H7 or immunoglobulin G controls (MAb EM-7G1) (1 µg/ml) for 30 min at room temperature, washed and added to Caco-2 cells. The number of bacterial cells that adhered to the monolayer were enumerated. Percent adhesion was calculated relative to the inoculums that were added to the Caco-2 cells for the adhesion assay. Data are average (SD) of three independent experiments performed in duplicate. Bars marked with letters (a, b) indicate significant difference at *P*<0.05. (b) Representative Giemsa-stained Caco-2 cell monolayers showing visual evidence for qualitative adhesion characteristics of Lbp^WT^ and Lbp^LAP^ cells. Bar, 10 µm.

We also examined the adhesion and transepithelial translocation characteristics of recombinant Lbp^LAP^ in Caco-2 cells. The data showed a significant increase (*P* = 0.0009) in adhesion of Lbp^LAP^ compared to Lbp^WT^ (Fig 3a), demonstrating the involvement of LAP in adhesion. Giemsa staining of the Caco-2 cell monolayer also provided visual confirmation of qualitative increase in adhesion for Lbp^LAP^ cells (Fig 3b). LAP involvement was further verified by pre-treatment of Lbp^LAP^ cells with anti-LAP monoclonal antibody (MAb-H7) which reduced adhesion by 4.3% compared to antibody-untreated Lbp^LAP^ cells or cells treated with isotype immunoglobulin G control antibody (Fig 3a).

**Figure 4:**
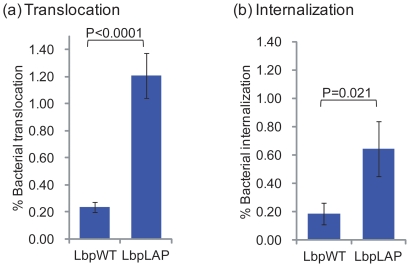
(a) Translocation and (b) internalization of recombinant *Lactobacillus paracasei* (Lbp^LAP^) and wild type *Lb. paracasei* (Lbp^WT^). In the translocation assay, Caco-2 cells were grown on transwell filter inserts for 10–12 days to differentiate and to reach confluence. Bacteria were added to the apical well of the insert and incubated for 2 h. Liquid from the basal well was removed and plated for bacterial enumeration. In the invasion assay, bacteria were added to Caco-2 cells at an MOE of 10:1/well in 24-well tissue culture plates and incubated for 1 h. After washing (3X), Caco-2 cells were incubated in D10F containing 50 µg/mL gentamicin, lysed using 0.1% Triton-X 100 and intracellular bacteria were enumerated following plating. The data are average (SD) of three independent experiments analyzed in duplicate.

Recently, we reported that LAP promotes *L. monocytogenes* translocation through epithelial cells using the paracellular route [21]. In this study, we examined whether Lbp^LAP^ had translocation ability similar to that of *L. monocytogenes*. Using a standard transwell setup, we showed that Lbp^LAP^ cells translocated through epithelial cell monolayers with greater efficiency—i.e., 5.1-fold (*P*<0.0001) higher—than that of Lbp^WT^ (Fig 4a). We also examined the internalization of Lbp^LAP^ by Caco-2 cells. Interestingly, Lbp^LAP^ cells were internalized at about a 3.5-fold higher level than that of the Lbp^WT^ (Fig 4b).

### Lbp^LAP^ Reduces *L. monocytogenes* Adhesion and Transepithelial Translocation through Caco-2 Cell Monolayers

**Figure 5:**
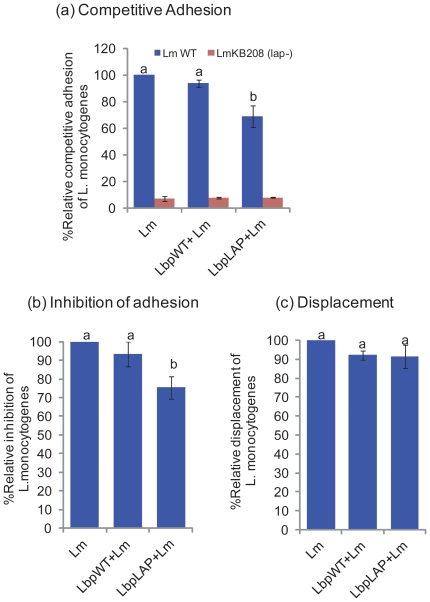
Competitive exclusion of *Listeria monocytogenes* (Lm) adhesion to Caco-2 cells by recombinant *Lactobacillus paracasei* (Lbp^LAP^), analyzed by three adhesion methods. (a) competitive adhesion: Caco-2 cells were exposed to wild type *Lb. paracasei* (Lbp^WT^) or Lbp^LAP^ with Lm simultaneously, (b) inhibition of adhesion: Caco-2 cells were pre-exposed to Lbp^WT^ or Lbp^LAP^ for 1 h before infection with Lm, and (c) displacement experiments: Caco-2 cells were infected with Lm for 1 h before Lbp^WT^ or Lbp^LAP^ addition. Adhesion of (Lm) alone to Caco-2 cells was presented as 100%. *Lap*-deficient LmKB208 was used as a negative control in the competitive adhesion assay. The data are average (SD) of three independent experiments performed in duplicate. Bars marked with letters (a, b) indicate significant difference at *P*<0.05.

We also investigated the ability of Lbp^LAP^ to reduce or prevent *L*. *monocytogenes* attachment to Caco-2 cells using the three competitive exclusion assays. In the competitive adhesion experiment, Caco-2 cells were exposed to Lbp^LAP^, Lbp^WT^, and *L. monocytogenes* for 1 h each before bacterial enumeration. In the competitive adhesion assay, adhesion of *L. monocytogenes* was reduced by 31.0% (Fig 5a), and in the inhibition of adhesion assay, reduction was 24.6% compared to that of *L. monocytogenes* alone (Fig 5b). No significant difference in displacement of *L. monocytogenes* occurred with Lbp^LAP^ (*P* = 0.3147; Fig 5c). Inhibition in adhesion of *lap*-deficient mutant *L. monocytogenes* KB208 by Lbp^LAP^ (negative control) was not observed. Overall, the recombinant strain effectively excluded *L. monocytogenes* when added before (inhibition of adhesion) or simultaneously (competitive adhesion) but not after *L. monocytogenes* has already adhered (displacement assay). We also monitored the adhesion of Lbp^LAP^ cells. These cells showed a 21.7% reduction in binding during competitive adhesion with *L. monocytogenes*, whereas no reduction occurred in the displacement assay; however, Lbp^LAP^ cell adhesion was significantly reduced after the inhibition assay (44.1% reduction) [52].

**Figure 6:**
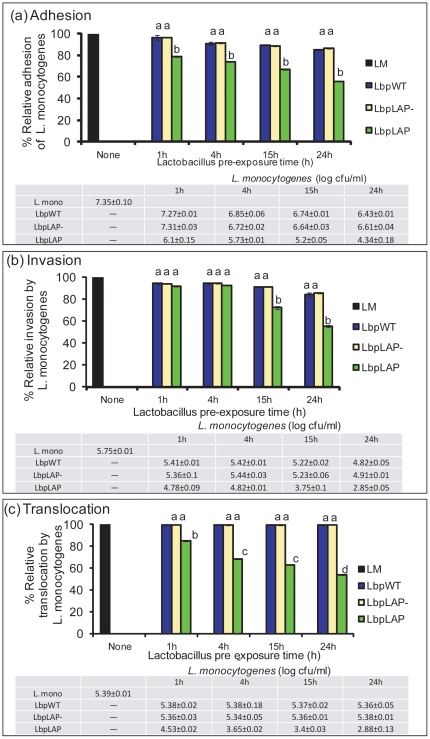
Inhibition of *Listeria monocytogenes* (Lm) adhesion, invasion, and transepithelial translocation by recombinant *Lactobacillus paracasei* (Lbp^LAP^). Caco-2 cells were exposed to Lbp^LAP^, Lbp^LAP-^ (vector without LAP insert) or wild type (Lbp^WT^) for 1, 4, 15, and 24 h before infection with Lm for 1 h in (a) adhesion and (b) invasion experiments, and 2 h for (c) transepithelial translocation experiments. Data are averages of three experiments run in triplicate. Bars marked with letters (a, b, c, d) are significantly different at *P*<0.05. Table below each graph shows average log Lm counts (SD) for each treatment.

Using the competitive adhesion assay, we determined the effect of Lbp^LAP^ cell pre-exposure on Caco-2 cells for 1, 4, 15, and 24 h and the reduction of *L. monocytogenes* infection—i.e., adhesion, invasion, and transepithelial translocation. The data showed that Lbp^LAP^ cells reduced *L. monocytogenes* adhesion by 21%, 26%, 33%, and 44%, respectively, whereas Lbp^WT^ exposure resulted in only a 3.5%-14.6% reduction during the same period (Fig 6a). Invasion experiment showed that Lbp^LAP^ reduced *L. monocytogenes* invasion by 8.3%, 7.3%, 27.6%, and 44.7%, respectively (Fig 6b). Transepithelial translocation experiments demonstrated highly significant effects against *L. monocytogenes*: Lbp^LAP^ reduced *L. monocytogenes* translocation by 15.3%, 31.8%, 36.8%, and 46.3% in 1, 4, 15, and 24 h, respectively (Fig 6c), whereas Lbp^WT^ had no significant effect. In these experiments, a vector control, devoid of *lap* insert (Lbp^LAP-^) was included to rule out the involvement of any plasmid encoded proteins (pLP401-T) that may exert protective effect against *L. monocytogenes* (Fig 6). Together, these data indicate that increased preoccupation of Hsp60 on Caco-2 cells by growing Lbp^LAP^ cells overtime significantly (*P*<0.05) reduced *L. monocytogenes* adhesion, invasion, and translocation though epithelial barriers.

### Lbp^LAP^ Reduces *L. monocytogenes*-Induced Tight Junction Permeability

*L. monocytogenes* may alter tight junction permeability to allow for its own translocation through the epithelial barrier [21]. Hence, we monitored Caco-2 tight junction integrity using the well-established dextran fluorescein isothiocyanate (Dextran^FITC^) permeability assay. After infection with *L. monocytogenes* for 2 h, about 2.6% of the apical Dextran^FITC^ was recovered from the basolateral chamber, indicating a compromise in tight junction integrity. In contrast, pre-exposure of Caco-2 monolayers to Lbp^LAP^ for 1–24 h before *L. monocytogenes* infection reduced Dextran^FITC^ recovery to 0.3% or less (Table 2), a level equivalent to that from uninfected Caco-2 cells. These data demonstrated that Lbp^LAP^ can protect Caco-2 cells from *L. monocytogenes*-mediated cell damage and tight junction compromise. Likewise, we monitored tight junction integrity by measuring transepithelial electrical resistance (TEER; Table 3). Percent change in TEER values for Caco-2 cells pre-exposed to Lbp^WT^ followed by 2 h of treatment with *L. monocytogenes* varied from 8.8% to 14.5%; however, values for Lbp^LAP^-treated cells followed by *L. monocytogenes* infection was only 1.4% – 6.4%. These data confirm the ability of Lbp^LAP^ to prevent *L. monocytogenes* translocation through epithelial cell barriers, possibly by maintaining tight junction integrity (see Fig 6).

**Table 2:** Tight junction integrity analysis with Dextran^FITC^ permeability assays.

Treatment	% Apical Dextran^FITC^ recovered in bottom well after Caco-2 cells were pretreated with *Lactobacillus paracasei* for variable time periods followed by *Listeria monocytogenes* treatment for 2 h (Mean [SE])^a^			
	1 h	4 h	15 h	24 h
*Lb. paracasei* WT (Lbp^WT^)	2.11±0.04	2.28±0.05	2.56±0.07	2.54±0.12
*Lb. paracasei* LAP (Lbp^LAP^)	0.09±0.01	0.32±0.02	0.34±0.001	0.34±0.01
Fold-change	24.8	7.1	7.5	7.5

a Caco-2 cells monolayers were grown in transwell inserts and treated with wild type (WT) or *Listeria* adhesion protein (LAP)-expressing *Lb. paracasei* for 1, 4, 15, and 24 h, then treated with *L. monocytogenes* for 2 h. Tight junction integrity of Caco-2 cells was monitored with Dextran^FITC^ translocation across the membrane. Dextran^FITC^ recovery after *L. monocytogenes* was 2.68±0.03%. Values are averages of three experiments analyzed in triplicate and are significantly different between Lbp^WT^ and Lbp^LAP^ at all time points (*P*<0.05).

**Table 3:** Caco-2 cell permeability analysis using transepithelial electrical resistance (TEER).

Treatment	Exposure time (h)	TEER (Mean Ω/cm^2^ [SE])^a^		
		Before exposure to *Listeria monocytogenes*	After exposure to *L. monocytogenes* (2 h)	% Change
*Lactobacillus paracasei* WT (Lbp^WT^)	1 h	268.6±3.9	244.9±4.7	8.8
	4 h	269.9±2.9	239.9±2.1	10.8
	15 h	265.5±3.3	226.9±2.2	14.5
	24 h	271.4±2.4	232.9±3.1	14.2
*Lb. paracasei* LAP (Lbp^LAP^)	1 h	266.5±3.4	262.9±3.1	1.4
	4 h	267.1±3.5	261.5±4.0	2.1
	15 h	263.9±1.5	252.5±0.8	4.3
	24 h	268.7±4.1	251.5±3.6	6.4

a Caco-2 cells monolayers were grown in transwell inserts and treated with wild type (WT) or *Listeria* adhesion protein (LAP)-expressing *Lb. paracasei* for 1, 4, 15, and 24 h, then treated with *L. monocytogenes* for 2 h. TEER measurements before and after *L. monocytogenes* treatment alone were 279.40±1.19 and 243.57±1.20, respectively. Values are averages of two experiments analyzed in triplicate and are significantly different between Lbp^WT^ and Lbp^LAP^ at all time points (*P*<0.05). % Change was calculated as 1 – TEER_after_ ÷ TEER_before_ ×100.

### Lbp^LAP^ Reduces *L. monocytogenes*-Induced Cell Cytotoxicity

**Figure 7:**
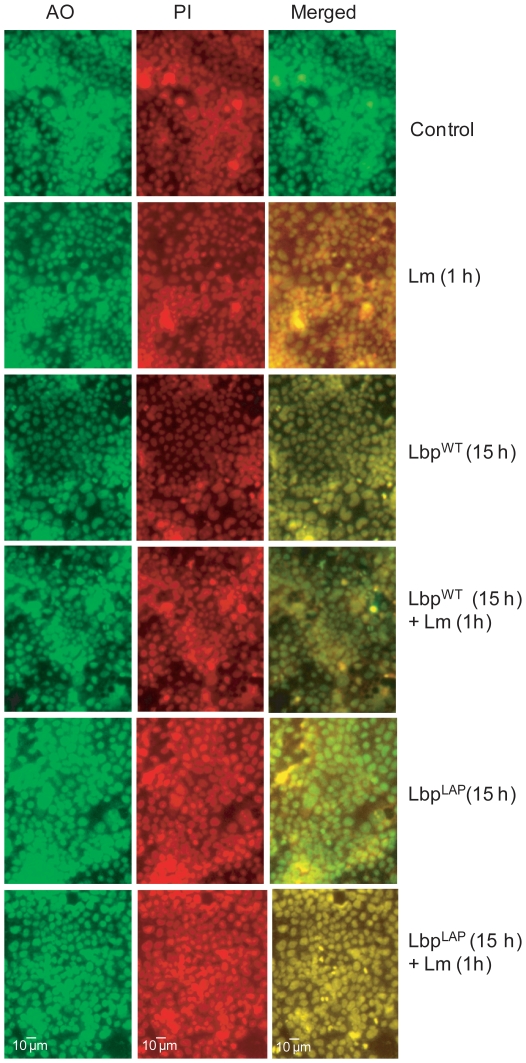
Microscopic analysis of protection of Caco-2 cells from *Listeria monocytogenes* (Lm)-mediated damage by recombinant *Lb. paracasei* (Lbp^LAP^). Caco-2 cells pre-exposed to wild type *Lb. paracasei* (Lbp^WT^) or Lbp^LAP^ for 15 h before infection with Lm for 1 h were stained with a mixture of acridine orange (green) for live cells and propidium iodide (red) for dead cells. Orange-red cells in the merged picture indicate dead or dying cells. Bar, 10 µm.

*L. monocytogenes* induces severe cell cytotoxicity in mammalian cells [53]. We examined whether Lbp^LAP^ could protect Caco-2 cells from this cytotoxicity. Lactate dehydrogenase assay indicated that Lbp^LAP^ reduced *L. monocytogenes*-mediated cytotoxicity by 99.8% after 1 h of pre-exposure, 88.8% after 4 h, 80% after 15 h, and 79% after 24 h, whereas Lbp^WT^ demonstrated no discernable protective effects (Table 4). Reduced Lbp^LAP^-mediated protection after 15 and 24 h of pre-exposure may be due to the overgrowth of Lbp^LAP^ and consequent production of metabolic by-products with adverse effects on Caco-2 cells, which make them more vulnerable to *L. monocytogenes*-mediated cell damage. Under *in vivo* conditions, these by-products would most likely be processed by luminal cells or natural microflora [54]. Reduced cytotoxicity was also verified with live and dead staining of Caco-2 cells using acridine orange (AO) and propidium iodide (PI). *L. monocytogenes*-infected Caco-2 cells pretreated with and without Lbp^WT^ for 15 h appeared orange-red, indicating that the majority of cells were either dead or their cell membranes were severely compromised. When the Caco-2 cells were pre-exposed to Lbp^LAP^ before *L. monocytogenes* infection, however, they appeared bright green, indicating that they were similar to uninfected controls (Fig 7).

**Table 4:** Cytotoxicity of *Listeria monocytogenes* on Caco-2 cells pretreated with *Lactobacillus paracasei.*

Treatment	% Cytotoxicity induced by *L. monocytogenes* to Caco-2 cells pretreated with *Lb. paracasei* for variable time periods (Mean [SE])^a^			
	1 h	4 h	15 h	24 h
*Lb. paracasei* WT (Lbp^WT^)	56.9±0.14	59.0±0.7	61.6±0.8	65.3±0.9
*Lb. paracasei* LAP (Lbp^LAP^)	0.09±0.02	7.4±1.5	12.7±0.3	13.7±0.6
% Protection	99.8	88.8	80	79

a *Lb. paracasei* cultures were added to Caco-2 cells at a multiplicity of exposure (MOE) of 10∶1 for 1, 4, 15, and 24 h before infection with *L. monocytogenes* (MOI of 10∶1) for 1 h. Cytotoxicity value for *L. monocytogenes* alone was 66.21±3.1. Values are averages of three experiments analyzed in triplicate and are significantly different between Lbp^WT^ and Lbp^LAP^ at all time points (*P*<0.05).

## Discussion

Immunocompromised populations such as pregnant women and their fetuses, infants, the elderly, HIV-infected patients, and patients receiving chemotherapy are most vulnerable to infectious diseases [1], [55], [56]. Increasing concerns about antibiotic resistance [26], the emergence of superbugs [5], and the lack of targeted treatments has created renewed interest in using probiotic bacteria against these diseases [3], [22], [23]. In this respect, recombinant probiotics expressing genes that are required for pathogen adhesion and colonization [3] are even more attractive targets because these steps are primary and critical for pathogenesis. In *L. monocytogenes* pathogenesis, adhesion, invasion, and translocation of intestinal epithelial barriers are crucial during the gastrointestinal phase of infection [7], [8]. Blocking the initial adhesion/invasion of *L. monocytogenes* would be a rational strategy for controlling infection. Recently, we have shown that LAP promotes *L. monocytogenes* translocation through epithelial barriers via the paracellular route [21]. In addition, it induces increased Hsp60 production, allowing for greater secondary infection with *L. monocytogenes* [21].

Probiotic bacteria are considered safe and have health benefits. Probiotics are also known to exclude enteric pathogens or neutralize toxins [4], [57]. Genetically engineered probiotics have been shown to be effective against *E. coli* heat-labile toxin (LT) and cholera toxin [42], [43], *Helicobacter pylori* [44], HIV [45], and *Salmonella enterica* infections [46]. Probiotics also prevent pathogen infections by increased defensin production, induction of anti-inflammatory responses and suppression of pro-inflammatory cytokines, increased production of short-chain fatty acids and improved epithelial tight junction barrier function [28]-[31]. Thus, we were interested in generating recombinant probiotic bacteria expressing LAP for potential control of *L. monocytogenes* infection.

Initially, the adhesion ability of LAB to Caco-2 cells was screened to find strains useful for LAP expression. Although adhesion profiles varied widely among LAB strains, overall, it was within the range of previously reported values [58], [59]. From the LAB strains tested, *Lb. rhamnosus*, *Lb*. *acidophilus*, and *Lb. paracasei*, all with different adhesion abilities, were selected. The rationale for choosing three species with broad range of adhesion properties was that competitive exclusion depends not only on the adhesion ability of a probiotic strain [60] but also on properties such as acid and antimicrobial compound production [36], [37], [61]. Surprisingly, none of the three species significantly excluded *L. monocytogenes* adhesion. Competitive exclusion against other pathogens such as *E. coli* O157:H7, *Salmonella typhimurium*, *Campylobacter jejuni*, *Shigella boydii*, and *Staphylococcus aureus* was successful using lactobacilli [62]–[64]. However, *L. monocytogenes* had been considered a difficult organism to inhibit using *Lactobacillus* species. Botes et al. [65] have prevented *L. monocytogenes* Scott A invasion using bacterial cells and the SN of *Enterococcus mundtii* and *Lb*. *plantarum*, but no effect on adhesion was observed during competitive exclusion assays. Another study has shown no significant effect on *L*. *monocytogenes* inhibition by *Lb. acidophilus* and *Lb. casei*, whereas *Lb. rhamnosus* GG partially blocked *L. monocytogenes* adhesion [59]. Collado et al. [10] have shown that bifidobacterium has a greater ability to displace *L. monocytogenes* than to inhibit it, but they did not observe significant reduction in adhesion in displacement and inhibition experiments. Coconnier et al. [33] have shown that *Lb. acidophilus* inhibits *L. monocytogenes*; however, the effect was dose dependent and more than 10^9^ cfu/mL of *Lb. acidophilus* was needed.

Among the lactobacilli chosen for our study, *Lb. paracasei* was used as the host for generation of the recombinant strain because the *Lactobacillus* expression vector pLP401-T delivers protein effectively due to the presence of a secretion signal, and the leader sequence of CW proteinase from *Lb. paracasei* [66]. The fusion of these sequences with heterologous genes permits secretion and surface association of heterologous proteins to the peptidoglycan via anchor encoding sequence, *prtP* from *Lb. casei* [50]. This has been also used to express tetanus toxin fragment C [49], and single-chain Fv antibody fragment against *Streptococcus mutans* [67]. Before initiating the cloning experiment, we verified if *Lb. rhamnosus*, *Lb. acidophilus*, and *Lb. paracasei* would interact with Hsp60, because they also carry a LAP homolog (Table S1); however, a magnetic bead binding experiment (Fig S3) and a microfluidic biochip experiment [68] revealed no apparent interaction of these lactobacilli with purified human Hsp60 protein. Western blot analysis also revealed no apparent reaction of anti-LAP antibody with protein fractions from Lbp^WT^ (see Fig 2). We then successfully expressed LAP in *Lb. paracasei* and surface-associated LAP from Lbp^LAP^ interacted strongly with Hsp60 in a magnetic bead binding experiment (Fig 2).

Additionally, Lbp^LAP^ also showed greater adhesion, internalization and translocation than that of Lbp^WT^ to Caco-2 cells. LAP is not known to promote invasion [21] but the increased association between Lbp^LAP^ and epithelial cells might have influenced increased uptake by the latter, a phenomenon that has frequently been reported for nonpathogens [69], [70]. Nevertheless, these results provide strong evidence that increased translocation be mediated by the specific binding of LAP to Hsp60. *Lactococcus lactis* expressing InlA of *L. monocytogenes* or fibronectin binding protein from *S. aureus* showed internalization 1000 times higher than that of the WT [71]. These recombinant bacteria also transferred plasmids carrying foreign genes into Caco-2 cells, suggesting potential use of these strains for DNA delivery. Our recombinant Lbp^LAP^ strain also shows potential for delivering foreign proteins to protect hosts against listeriosis or other infections, a property currently under investigation.

In future, we would like to determine the fate of these translocated bacteria in the lamina propria in *in vivo* animal models following oral administration [28], [31] and determine immune response, if any, to this protein. Since, *L. monocytogenes* infection is fatal in immunocompromised hosts we foresee that the LAP-expressing probiotics can be taken orally as a dietary supplement in a regular basis by this population during the period of need such as women during pregnancy, organ transplant patients receiving immunosuppressive drugs, cancer patients receiving chemotherapy or the elderly.

Increased translocation of probiotic bacteria may raise potential health concerns, particularly in immunocompromised patients; a few cases of sepsis related to translocated *Lactobacillus* have been reported in these patients [72]. However, translocated *Lactobacilli* are rapidly eliminated by the host immune system and thus may not be found even when administered in higher doses [73]. Acceptable daily intake of probiotics is 35 g/day for a person weighing 70 kg, which is much higher than what is normally consumed and suggests very low risk of infection [72]. Even though probiotics are considered safe, recombinant strains must be thoroughly evaluated *in vivo* for toxicity before use [48]. Competitive adhesion experiments revealed that Lbp^LAP^ was able to reduce the adhesion of *L. monocytogenes* only when it was added simultaneously or sequentially with *L. monocytogenes*; however, the recombinant strain was unable to displace adhered *L. monocytogenes*. (see Fig 5). The competition was presumably based on interactions with Hsp60 expressed on the surface of Caco-2 cells. It is difficult to displace attached pathogens unless the bacteria are detached and the recombinant *Lactobacillus* is allowed to bind to the receptor to hinder pathogen adhesion. Lee et al. [63] have examined competitive exclusion of pathogens such as *E. coli* O157:H7, *Salmonella enteritidis*, and *S. typhimurium* by *Lb. rhamnosus* GG and have suggested that displacement was relatively slow and more difficult, but prolonged exposure of *Lactobacillus* could remove adhered *L. monocytogenes* from Caco-2 cells. The interaction between Hsp60 and LAP is strong, with a binding affinity of 1.68×10^−8^ M [13]. Thus, prolonged exposure to *Lactobacillus* expressing LAP may not result in displacement of adhered *L. monocytogenes*. Moreover, during the displacement experiment, the pathogen may have already proceeded to the next step of the infection process—i.e., invasion or transepithelial translocation—before the probiotics had an opportunity to displace them.

Using the competitive adhesion assay, a prolonged exposure (1–24 h) of Caco-2 cells to Lbp^LAP^ cells was tested to show increased inhibition, with the highest reduction noticed after a 24-h pre-exposure. In addition to a reduction in adhesion and invasion, prolonged exposure to the recombinant probiotic also significantly reduced the transepithelial translocation of *L. monocytogenes*. Therefore, prolonged exposure to the probiotic (Lbp^LAP^) may be necessary to prevent *L. monocytogenes* infection. These data indicate that Lbp^LAP^ may protect Caco-2 cells via an unknown mechanism currently under investigation. We speculate that Lbp^LAP^ increases tight junction integrity by suppressing the production of TNF-α and interferon-γ as part of a “leak pathway” and subsequently regulates cytoskeletal protein expressions involved in maintaining tight junction integrity [9], [74]. Our preliminary unpublished data show that purified recombinant LAP was also able to increase tight junction permeability in Caco-2 cells allowing increased translocation of a nonpathogenic strain of *E. coli* suggesting that recombinant LAP expressing probiotics bacteria cells are required to provide physical barrier against pathogen invasion and translocation.

Genetically modified lactic acid bacteria are becoming attractive vehicles for delivering functional proteins to the mucosal tissues via oral or intranasal route to induce mucosal immunity against infectious agents [40]. Thus, the use of a recombinant probiotic carrying the LAP (Lbp^LAP^) could be considered an oral vaccine to help reduce *L. monocytogenes* infection in high-risk populations. Furthermore, the application of such a recombinant probiotic bacterium would have a two-fold advantage: direct antimicrobial effect against the target pathogen through the expression of the foreign gene and indirect general health benefits through consumption of probiotics.

## Materials and Methods

### Bacterial Strains, Plasmids, and Growth Conditions

Bacterial strains and plasmids used in this study are listed in Table 1. All *Listeria* species were grown in brain heart infusion (BHI, Becton Dickinson, Sparks, MD) or Luria-Bertani broth (LB, 0.5% NaCl, 1% tryptone peptone, and 0.5% yeast extract) at 37°C for 16 to 18 h. All lactic acid bacteria except *Lactococcus lactis* were cultured in deMan Rogosa Sharpe broth (MRS, Becton Dickinson) at 37°C for 18–20 h. *Lc. lactis* strains were grown in M17 broth (Becton Dickinson). *Lb. rhamnosus, Lb. paracasei*, and *Lb. gasseri* were grown at 37°C under anaerobic conditions. The *lap*-deficient mutant *L*. *monocytogenes* strain KB208 was grown in BHI or LB with erythromycin (5 µg/mL) at 42°C. pLP401T [50] was used for LAP expression in *Lb. paracasei* and was grown in appropriate media with ampicillin (50 µg/mL) for *E*. *coli*, and erythromycin (2 µg/mL) for *Lb. paracasei*. To induce expression of LAP in recombinant *Lb. paracasei*, the bacterium was grown in modified MRS (1% w/v proteose peptone, 0.5% w/v yeast extract, 0.2% w/v meat extract, 0.1% v/v Tween 80, 37 mM C_2_H_3_NaO_2_, 0.8 mM MgSO_4_, 0.24 mM MnSO_4_, 8.8 mM C_6_H_14_N_2_O_7_ in 0.1 M potassium phosphate buffer, pH 7.0) supplemented with mannitol (1% w/v).

### Construction of Lbp^LAP^

Genomic DNA from *L. monocytogenes* F4244 was purified and the *lap* gene was amplified from genomic DNA with polymerase chain reaction using primers LAPLmN-F 5′-GACCATGGATGGCAATTAAAGAAAATG-3′ and LAPLmX-R5′-GACTCGAGTCAAACACCTTTGTAAG-3′ (Integrated DNA Technologies, Coralville, IA). The amplified DNA was cloned into pGEM-T Easy Vector (Promega, Madison, WI) and designated pGEM-LAPLm. Lactobacilli expression vector, pLP401-T was used to express LAP in *Lb. paracasei* (48; Fig. 2a). This vector has been shown to be efficient for heterologous protein delivery by lactobacilli, owing to the presence of a secretion signal and the leader sequence of cell wall proteinase (*prtP*) from *Lb. casei* [66]. This gene sequence codes for the secretion and surface association of heterologous proteins to the peptidoglycan [49]. The plasmid was digested with *Nco*I and *Xho*I, inserted into expression vector pLP401T, and designated pLP401T-LAP. To remove the terminator, which stabilizes the plasmid in *E. coli* [50], pLP401T-LAP was digested with *Not*I and pLP401T-LAP was obtained via self-ligation. Self-ligated pLP401T-LAP was transformed into *Lb*. *paracasei* by electroporation. Competent *Lb*. *paracasei* cells were prepared with incubation of 2% culture in fresh MRS broth containing 0.5% sucrose and 0.5% glycine at 37°C until OD_600_ reached to 0.5 ∼ 0.8. The cells were harvested (3,900×g for 5 min at 4°C), washed twice with washing buffer (0.5 M sucrose, 10% glycerol) and collected. Then the cells were resuspended in the same washing buffer and stored at – 80°C. For electroporation, 50 µl of competent cells mixed with 0.5 µg of purified plasmid DNA in an ice cold cuvette with a 2-mm electrode gap. The electric pulse was delivered by the Gene Pulser XcellTM electroporation system (Bio-Rad, Richmond, CA) using the following parameter settings: 1.5 kV, 200Ω and 25 µF. After electroporation, competent cells were recovered in 1 ml of MRS containing 0.5 M sucrose, 20 mM MgCl_2_, 2 mM CaCl_2_ at 37°C for 2 h in water bath. Transformants were selected using MRS agar containing 2 µg/mL of erythromycin [52]. Similarly, another recombinant strain was generated carrying the pLP401-T plasmid with no LAP insert to be used as a vector control (Lbp^LAP-^). Identity of recombinant and WT *Lb. paracasei* strains were confirmed by ribotyping using an automated RiboPrinter® (DuPont Qualicon, Wilmington, DE). Protein expression in recombinant strains was confirmed with Western blot analysis.

### Analysis of LAP Expression by *Lb. paracasei*

LAP expression in SN, CW, and intracellular fractions was analyzed. SN was collected from centrifuged culture (7,000×g for 10 min at 4°C) and the pellet was retained for preparation of CW and intracellular proteins. The SN was filtered (0.22-µm filter), precipitated with 10% trichloroacetic acid for 40 min on ice, and centrifuged (14,000×g at 4°C for 10 min). The pellet was resuspended in ice-cold acetone and centrifuged. The remaining acetone was evaporated, and the pellet was resuspended in alkaline rehydration buffer (100 mM Tris-base, 3% SDS, 3 mM dithiothreitol, pH 11), boiled for 5 min, and stored at −20°C.

For the CW protein fraction, the pellet was resuspended in 5 M LiCl with 5 mM EDTA and incubated for 30 min in a water bath at 37°C. The suspension was centrifuged (13,000×g at 4°C for 5 min) and the SN was filtered (0.45-µm filter). The sample was dialyzed using ultrapure water supplemented with 5 mM EDTA and stored at −20°C.

The pellet from the CW protein preparation was used for intracellular protein isolation. It was resuspended in the sample solvent (5% SDS, 0.5% β-mercaptoethanol, 1.5% Tris, pH 7.0) and sonicated on ice for 5–7 cycles of 15 sec each using a Sonifier 150D (Branson, Niantic, CT). The sample was centrifuged and the SN was collected and stored at −20°C. SN and CW protein preparations were also tested with a PepC assay [75] to rule out contamination with intracellular or membrane proteins.

Proteins were quantified using the bicinchoninic acid method (Pierce, Rockford, IL) and equivalent amounts of protein (40 µg of each fraction) were separated using SDS polyacrylamide gel electrophoresis (7.5% acrylamide) gel. The proteins were transferred to an Immobilon-P membrane (Millipore, Billerica, MA) and immunoprobed with anti-LAP antibody MAb-H7 (1.0 µg/mL) and horseradish peroxidase-coupled anti-mouse antibody (0.2 µg/mL; Jackson Immuno Research, West Grove, PA). The membranes were developed with an enhanced chemiluminescence kit (Pierce).

LAP expression in recombinant probiotics was also determined by reacting 18-h grown bacterial cells first with MAb-H7 for 1 h followed by FITC-labeled anti-mouse monovalent secondary Fab fragment (diluted 1∶250 in phosphate-buffered saline [PBS]; Jackson Immuno Research) for 1 h and counterstained with Hoechst dye (0.5 µg/mL in PBS; Invitrogen) for nucleus staining. Cells were washed between antibody treatments with PBS containing 1% bovine serum albumen and examined under a fluorescence microscope (Leica, model DMLB, Wetzlar, Germany) equipped with SPOT software (version 4.6.4.2, Diagnostic Instruments, Sterling Heights, MI, USA).

### Analysis of Recombinant Probiotic Interaction with Hsp60-Coated Paramagnetic Beads

A magnetic bead capture method was used to analyze the interactions of surface-associated LAP on the recombinant probiotic with human Hsp60. Paramagnetic beads (MyOne™ Streptavidin C1 beads; average diameter, 1.0 µm; Invitrogen, Carlsbad, CA) were coated with biotinylated Hsp60 as described elsewhere [51]. Briefly, PBS-washed, overnight-grown bacterial cells (250 µL) were mixed gently with Hsp60-coated beads (20 µL) and incubated at 25°C for 1 h on a vortex mixer. The beads were removed using a magnetic particle concentrator (MPC-S; Invitrogen) and washed three times with PBS (20 mM, pH 7.0) and once with PBS containing 0.5% Tween 20. Captured bacteria were mechanically separated from the beads by vigorous vortexing; lactobacilli were quantified by plating on MRS agar (Becton Dickinson) and *Listeria* on modified oxford (MOX) agar (Becton Dickinson) plates after incubation at 37°C for 24–48 h.

### Caco-2 Cell Culture

Human colon carcinoma cell line Caco-2 (HTB37; American Type Culture Collection) was cultured in Dulbecco's modified Eagle's medium supplemented with 10% fetal bovine serum (D10F; Atlanta Biologicals, Norcross, GA). Passages of 20–35 were used for the experiments, and the cells were grown in 12- and 24-well plates at 37°C in the presence of 7% CO_2_ in a cell culture incubator for 10–12 days or until monolayers formed with no further visible differentiation.

### Adhesion Assays

The adhesion profiles of bacteria (10^6^ cfu/well) to Caco-2 cells (10^5^ cells/well) with multiplicity of exposure (MOE) of 10∶1 were analyzed using adhesion assays [13]. Adhered LAB was enumerated on MRS and *Listeria* on MOX agar plates. Additionally, bacterial adhesion to cell monolayers grown on glass coverslips was done by Giemsa staining followed by microscopic examination [76] to visualize bacterial attachment qualitatively.

To verify LAP-mediated binding, bacterial cells were also pretreated with anti-LAP antibody before use in the adhesion experiment [16]. As an immunoglobulin G isotype control, MAb EM-7G1 that reacts with a 66-kDa protein in *L. monocytogenes* was used.

### Invasion Assay

Invasion of bacteria was analyzed as previously described [76], [77]. Bacteria were added to Caco-2 cells at an MOE of 10∶1 and incubated for 1 h. The monolayers were washed with D10F, and an additional 1 h of incubation in D10F containing 50 µg/mL gentamicin followed. The cells were lysed with 0.1% Triton X-100 and plated for enumeration of internalized bacteria.

### Transepithelial Translocation Assay

Transepithelial bacterial translocation assay was performed as previously described [21], [78]. Briefly, Caco-2 cells were grown on transwell filter inserts (4-µm pore filter; Corning, Lowell, MA) for 10–12 days to reach confluence. Bacteria were added to the apical well of the insert and incubated for 2 h. Liquid from the basal well was removed, serially diluted if needed, and distributed onto plates for enumeration. TEER of Caco-2 cells before and after treatment was measured using a Millicell ERS system (Millipore, Billerica, MA).

### Competitive Exclusion of *L. monocytogenes* by LAB Strains

Competitive exclusion was determined using competitive adhesion, inhibition of adhesion, and displacement experiments [10]. A ratio of 10∶1 of *L. monocytogenes* or LAB strains to Caco-2 cells was used. (i) Competitive adhesion: *L. monocytogenes* and LAB strains were added simultaneously to Caco-2 cells and incubated for 1 h. To remove unbound bacteria, we washed the cells four times with Cell-PBS (137 mM NaCl, 5.4 mM KCl, 3.5 mM Na_2_HPO_4_, 4.4 mM NaH_2_PO_4_, 11 mM glucose, pH 7.2). Adherent bacteria were released by treatment with 0.1% Triton-X 100 in Cell-PBS and plated onto MOX for *L*. *monocytogenes* and MRS agar for LAB strains. (ii) Inhibition of adhesion: LAB strains were added to wells containing Caco-2 cells and incubated for 1 h. Unbound bacteria were removed by washing with D10F as above, and *L. monocytogenes* was then added and incubated for 1 h. The cells were then washed. Bound bacteria were released and plated as above. (iii) Displacement: *L. monocytogenes* were added to Caco-2 cells and incubated for 1 h. After washing with D10F, each LAB strain was added and incubated for 1 h. The cells were then washed. Bound bacteria were released and plated as above.

### Inhibition of *L. monocytogenes* Adhesion, Invasion, and Translocation by Lbp^LAP^

The ability of Lbp^LAP^ to inhibit *L. monocytogenes* adhesion, invasion, and translocation to Caco-2 cells was investigated as described elsewhere [79]. Lbp^LAP^ and Lbp^WT^ were added to each well and incubated for 1, 4, 15, or 24 h. Unbound bacteria were removed by washing with D10F, and *L. monocytogenes* was added (MOI; 10∶1) and incubated for 1 h for inhibition of adhesion and invasion experiments and 2 h for inhibition of translocation experiments. The cells were then washed. Bound bacteria were released by Triton-X treatment and plated as above. As a vector control, the recombinant Lbp^LAP-^ strain was used to rule out the involvement of any plasmid encoded proteins in protection against *L. monocytogenes* infection.

### Epithelial Tight Junction Integrity Analysis

Tight junction permeability of Caco-2 monolayers in transwell filter inserts (4-µm pore size; Corning) pre-exposed to probiotics for 1, 4, 15 and 24 h and infected with *L. monocytogenes* for 2 h was assessed by monitoring Dextran^FITC^ (M_r_ 3–5 kDa; Sigma) permeability as described elsewhere [80]. MOE for all bacterial strains was 10∶1. Dextran^FITC^ (1 mg/ml) was added to the transwell and incubated at 37°C for 1 h. Samples from the apical and basolateral chambers were collected and read in a SpectraMax Gemini EM fluorescent plate reader (Molecular Devices; Sunnyvale, CA). The data are expressed as percentages of the apical dextran recovered in the basal chamber.

### Cytotoxicity Assay and Fluorescence Microscopy

Caco-2 cell cytotoxicity was assessed using a lactate dehydrogenase cytotoxicity assay kit (Roche). Caco-2 cell viability was also assessed with live and dead staining of Caco-2 monolayers using a propidium iodide (PI; red, dead cell indicator) and acridine orange (AO, green, live cell indicator) mixture (PI; 100 µg/mL and AO; 20 µg/mL; Sigma) as described previously [81]. Stained cells were washed in Cell-PBS, fixed in methanol, and examined under a fluorescence microscope (Leica) equipped with SPOT software (Diagnostic Instruments) using green (for AO) and red (for PI) filters.

### Statistical Analysis

All experiments were repeated at least three times independently, and each set of experiments was performed in duplicate or triplicate. Statistical comparisons were carried out using analysis of variance (SAS 9.2, Cary, NC) and Tukey's multiple comparisons of means at *P*<0.05 to determine significant differences.

## Supporting Information

Figure S1 **Competitive exclusion analysis of** ***Listeria monocytogenes*** **by different** ***Lactobacillus*** **species to Caco-2 cells.** Three adhesion methods were used; (a) competitive adhesion, (b) inhibition of adhesion, and (c) displacement. First bar shows adhesion of *L. monocytogenes* to Caco-2 cells without pretreatment of LAB and presented as 100%. Tables (a1, b1, c1) under bar graph show percent adhesion values of *L. monocytogenes* with and without *Lb. rhamnosus, Lb. acidophilus* and *Lb. paracasei*. Also adhesion of each *Lactobacillus* species in the presence (w) and absence (w/o) of *L. monocytogenes* was shown. The data are average ± SD of three independent experiments analyzed in duplicate.
(TIF)

Figure S2 **Displacement of** ***Listeria monocytogenes*** **adhesion following pretreatment of Caco-2 cells with different (a) lactic acid bacterial (LAB) strains and (b) different ratios of** ***Lactobacillus rhamnosus*** **or** ***Lb. acidophilus*** **to** ***L. monocytogenes*** **.** First bar shows adhesion of *L. monocytogenes* to Caco-2 cells without pretreatment of LAB and presented as 100%. Other bars indicate relative adhesion rate of *L. monocytogenes* after addition of each LAB. The data are average ± SD of two independent experiments performed in triplicate.
(TIF)

Figure S3 **Binding (capture) analysis of different lactobacilli to Hsp60 coated paramagnetic beads.** First bar shows capture rate of *L. monocytogenes* to Hsp60-coated beads and presented as 100%. Other bars indicate relative capture rate for other bacteria. The data are average ± SD of two independent experiments performed in duplicate.
(TIF)

Figure S4 **Adhesion characteristics of bacteria to Caco-2 cells pretreated with anti-Hsp60 antibody.** (a) Adhesion of *L. monocytogenes* to Caco-2 cell monolayers that were pre-treated with anti-Hsp60 monoclonal antibody (1 µg/well for 1 h) or an isotype IgG control antibody (purified MAb C11E9 specific for *L. monocytogenes*) followed by exposure to Lbp^WT^, recombinant Lbp^LAP^, and a vector control, i,e., *Lb. paracasei* containing empty vector, pLP401-T without any LAP insert (Lbp^LAP-^) for 1 h. Adherent bacterial counts were determined by plating following lysis of cells using Triton-X 100. (b) Adhesion characteristics of Lbp^WT^ and Lbp^LAP^ to Caco-2 cells pretreated with anti-Hsp60 MAb or an isotype antibody MAb C11E9.
(TIF)

Table S1 **Sequence similarity between LAP, an alcohol acetaldehyde dehydrogenase (Aad) from** ***Listeria monocytogenes*** **and Lactobacilli.**
(DOCX)
