# Supplementary figures and images for: Recombinant Probiotic Expressing Listeria Adhesion Protein Attenuates Listeria monocytogenes Virulence In Vitro
Source: PLoS One. 2012 Jan 3;7(1):e29277. doi: 10.1371/journal.pone.0029277 (PMC3250429; doi:10.1371/journal.pone.0029277)

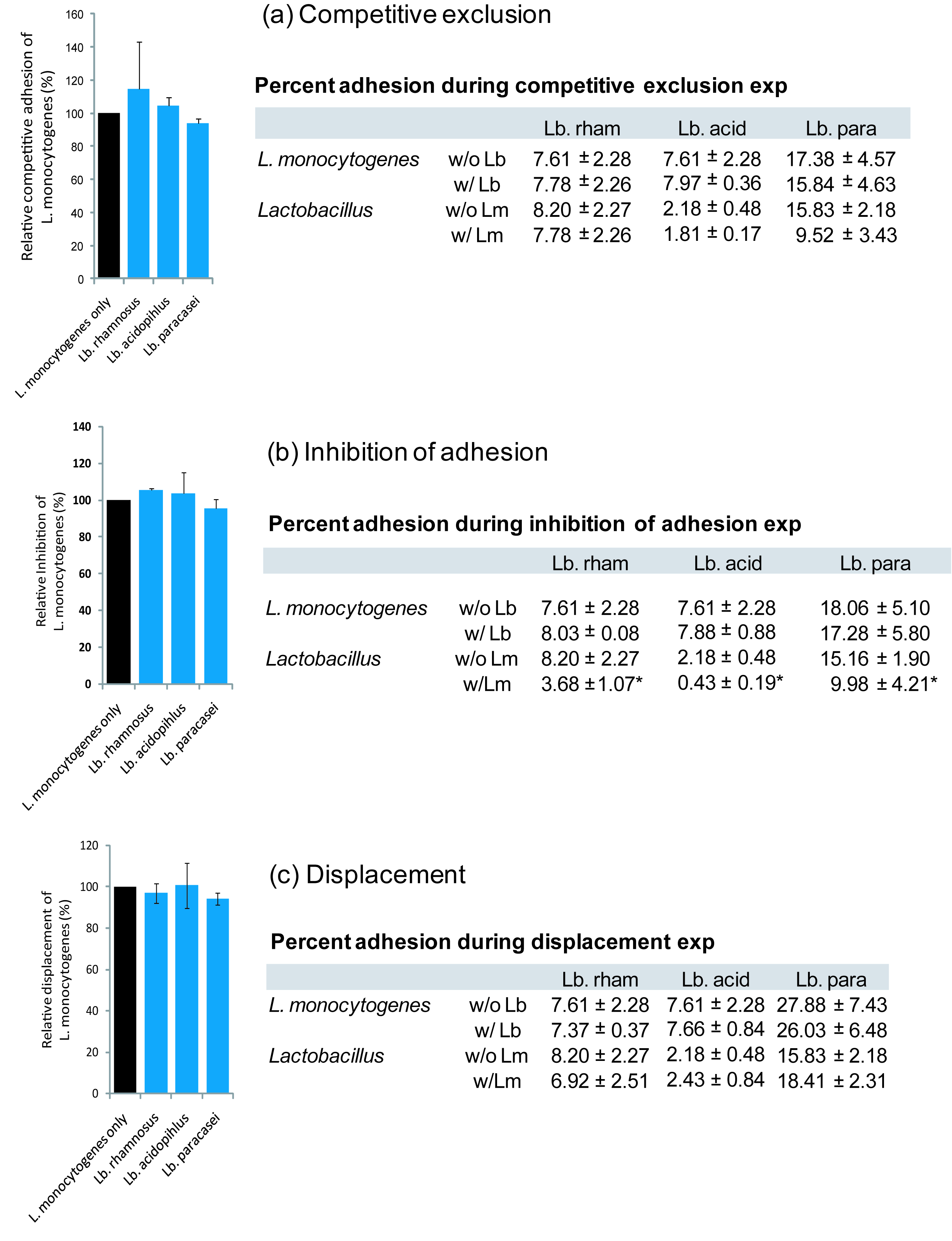

Supplement: Figure S1 — Competitive exclusion analysis of Listeria monocytogenes by different Lactobacillus species to Caco-2 cells. Three adhesion methods were used; (a) competitive adhesion, (b) inhibition of adhesion, and (c) displacement. First bar shows adhesion of L. monocytogenes to Caco-2 cells without pretreatment of LAB and presented as 100%. Tables (a1, b1, c1) under bar graph show percent adhesion values of L. monocytogenes with and without Lb. rhamnosus, Lb. acidophilus and Lb. paracasei. Also adhesion of each Lactobacillus species in the presence (w) and absence (w/o) of L. monocytogenes was shown. The data are average ± SD of three independent experiments analyzed in duplicate. (TIF) [file pone.0029277.s001.tif]

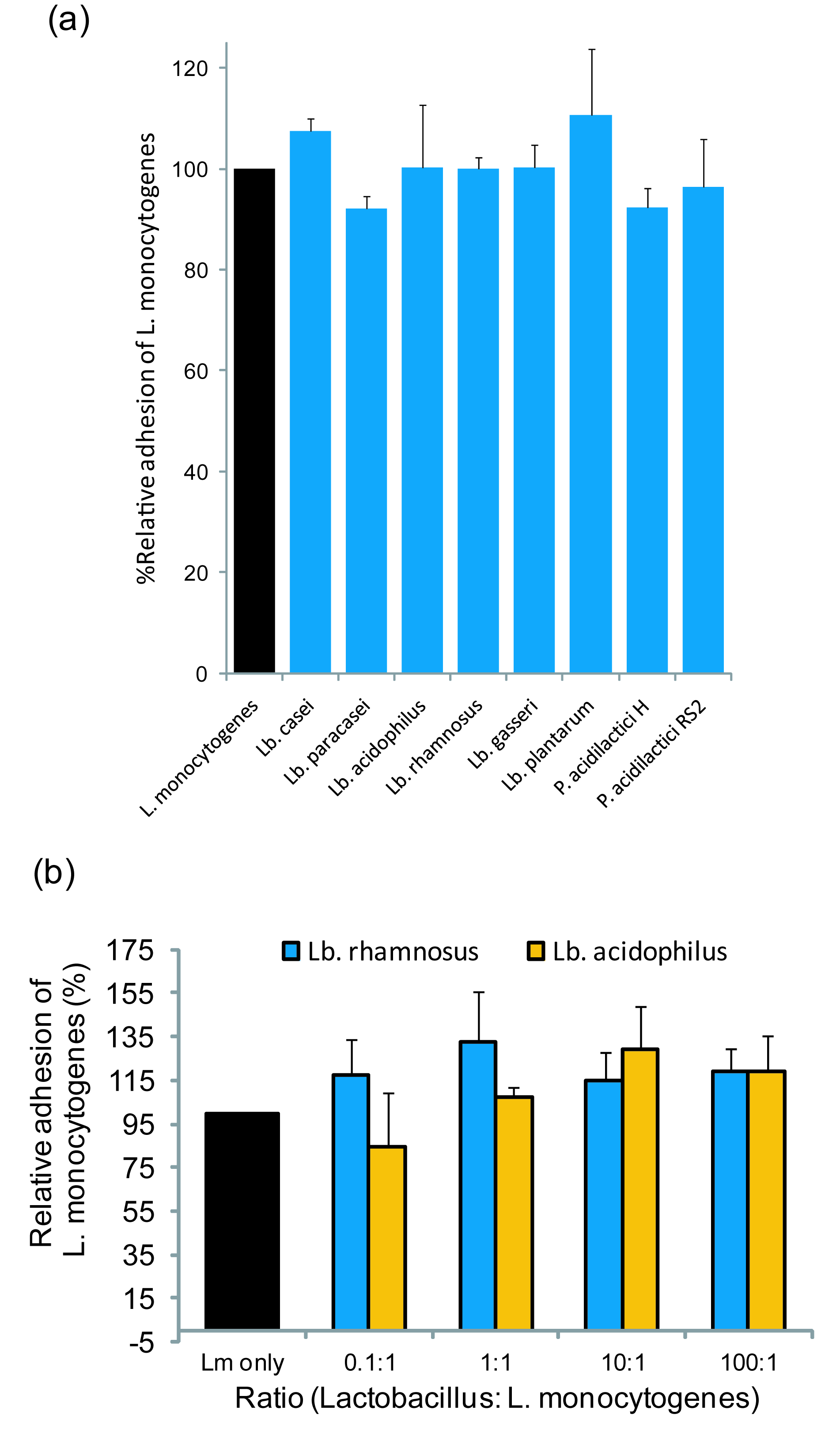

Supplement: Figure S2 — Displacement of Listeria monocytogenes adhesion following pretreatment of Caco-2 cells with different (a) lactic acid bacterial (LAB) strains and (b) different ratios of Lactobacillus rhamnosus or Lb. acidophilus to L. monocytogenes . First bar shows adhesion of L. monocytogenes to Caco-2 cells without pretreatment of LAB and presented as 100%. Other bars indicate relative adhesion rate of L. monocytogenes after addition of each LAB. The data are average ± SD of two independent experiments performed in triplicate. (TIF) [file pone.0029277.s002.tif]

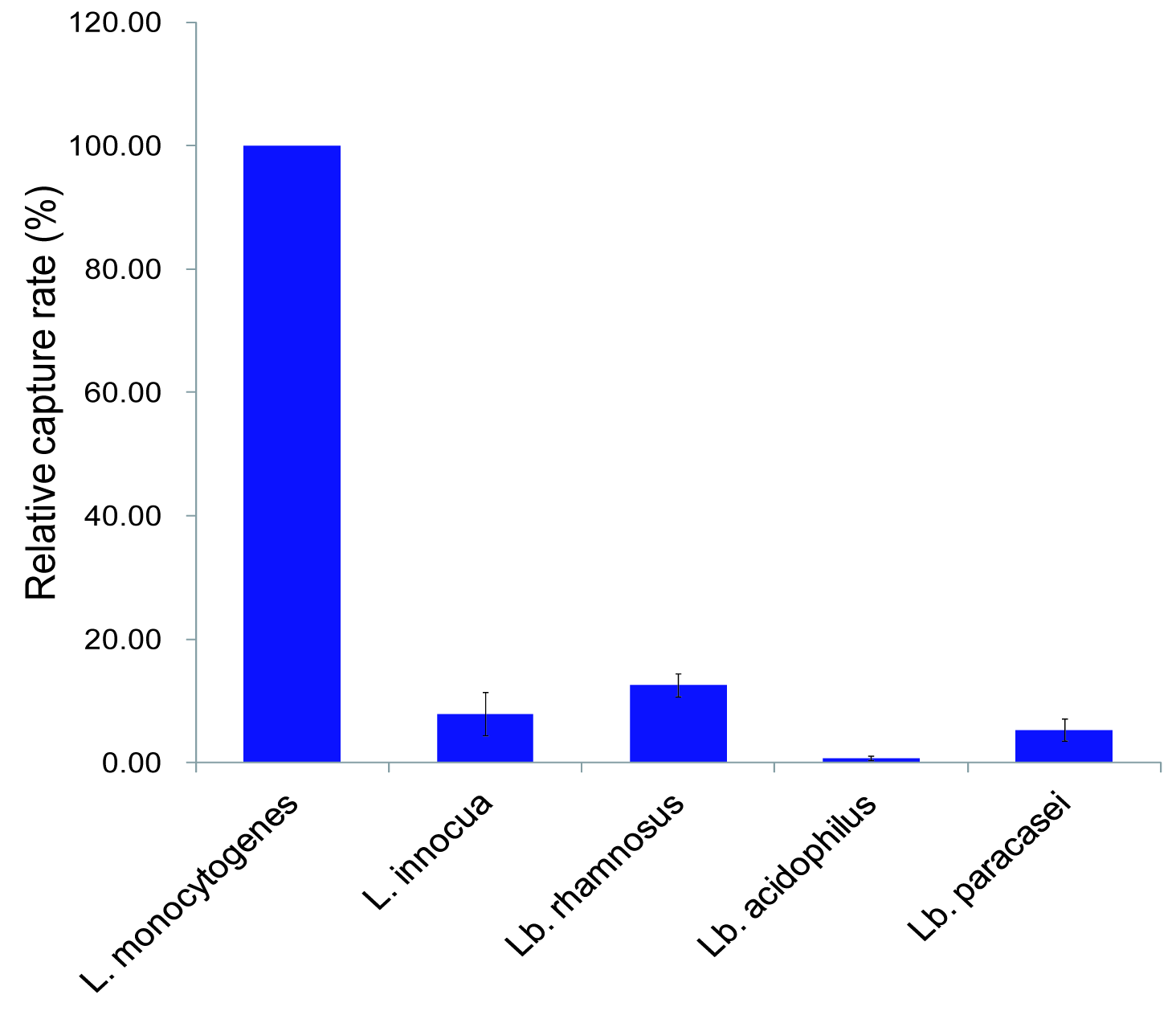

Supplement: Figure S3 — Binding (capture) analysis of different lactobacilli to Hsp60 coated paramagnetic beads. First bar shows capture rate of L. monocytogenes to Hsp60-coated beads and presented as 100%. Other bars indicate relative capture rate for other bacteria. The data are average ± SD of two independent experiments performed in duplicate. (TIF) [file pone.0029277.s003.tif]

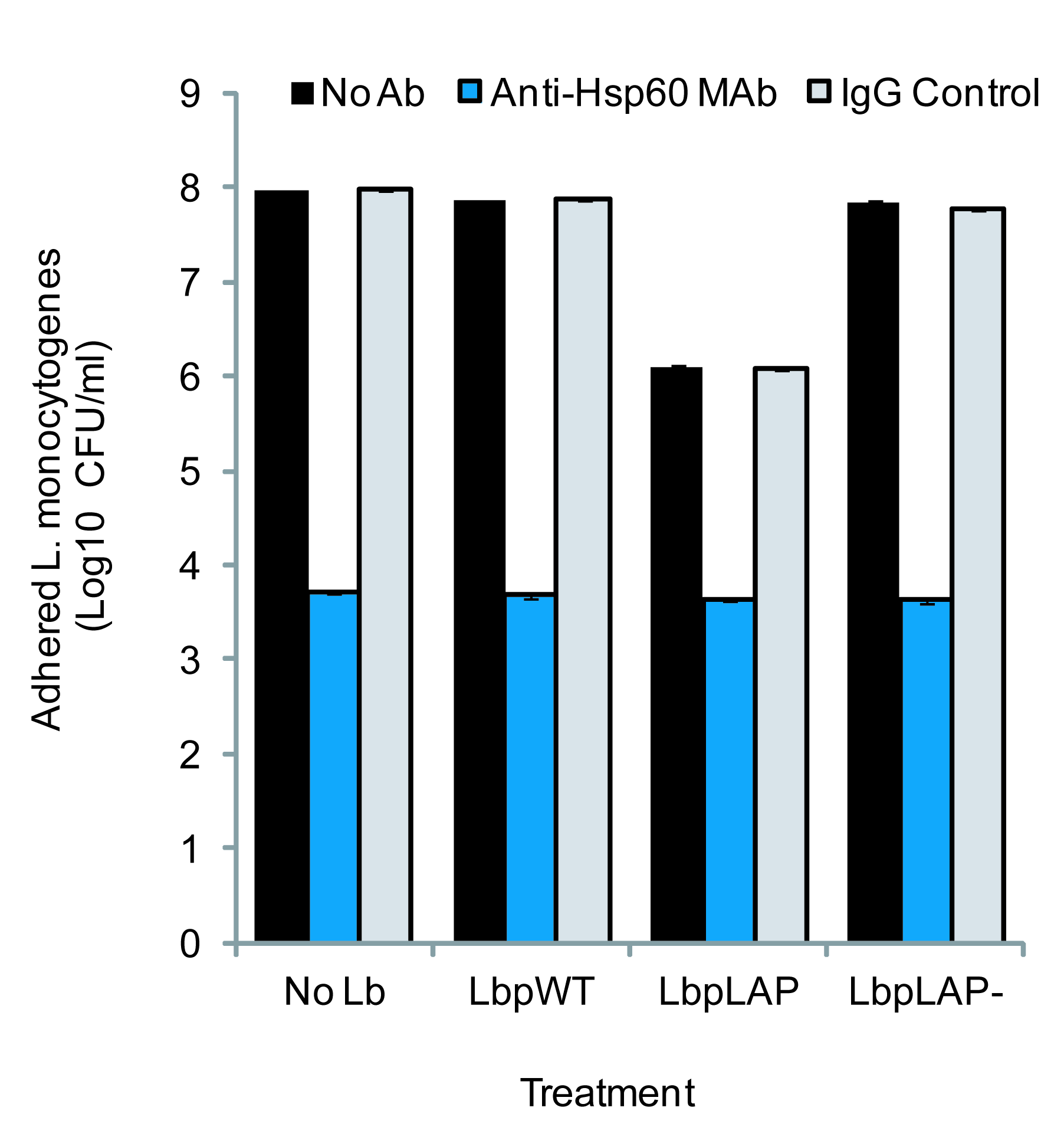

Supplement: Figure S4 — Adhesion characteristics of bacteria to Caco-2 cells pretreated with anti-Hsp60 antibody. (a) Adhesion of L. monocytogenes to Caco-2 cell monolayers that were pre-treated with anti-Hsp60 monoclonal antibody (1 µg/well for 1 h) or an isotype IgG control antibody (purified MAb C11E9 specific for L. monocytogenes) followed by exposure to LbpWT, recombinant LbpLAP, and a vector control, i,e., Lb. paracasei containing empty vector, pLP401-T without any LAP insert (LbpLAP-) for 1 h. Adherent bacterial counts were determined by plating following lysis of cells using Triton-X 100. (b) Adhesion characteristics of LbpWT and LbpLAP to Caco-2 cells pretreated with anti-Hsp60 MAb or an isotype antibody MAb C11E9. (TIF) [file pone.0029277.s004.tif]
