# Supplementary material for: Recombinant Probiotic Expressing Listeria Adhesion Protein Attenuates Listeria monocytogenes Virulence In Vitro
Source: PLoS One. 2012 Jan 3;7(1):e29277. doi: 10.1371/journal.pone.0029277 (PMC3250429; doi:10.1371/journal.pone.0029277)
Supplement: Table S1 — Sequence similarity between LAP, an alcohol acetaldehyde dehydrogenase (Aad) from Listeria monocytogenes and Lactobacilli. (DOCX) [file pone.0029277.s005.docx]

**Supporting Information**

**Table S1.** Sequence similarity between LAP, an alcohol acetaldehyde dehydrogenase (Aad) from *Listeria monocytogenes* and Lactobacilli.

| **Bacteria** | **NCBI accession** | **Identities (%)** | **Positives (%)** |
| --- | --- | --- | --- |
| *Lactobacillus rhamnosus* | ZP_03211500 | 60 % (528/871^a^) | 75 % (659/871) |
| *Lb. acidophilus* | YP_193379 | 50 % (443/878) | 67 % (593/878) |
| *Lb. paracasei* | ZP_04672734 | 60 % (525/872) | 75 % (661/872) |

^a^Number of identical or similar amino acids from total amino acids of Aad from *L. monocytogenes*
